# Outcomes of orbital malignancies treated with eye-sparing surgery and adjuvant particle radiotherapy: a retrospective study

**DOI:** 10.1186/s12885-019-5964-y

**Published:** 2019-08-06

**Authors:** Weixu Hu, Jiyi Hu, Jing Gao, Jing Yang, Xianxin Qiu, Lin Kong, Jiade J. Lu

**Affiliations:** 1 Department of Radiation Oncology, Shanghai Proton and Heavy Ion Center, Shanghai, China; 2 Shanghai Engineering Research Center of Proton and Heavy Ion Radiation Therapy, 4365 Kangxin Road, Pudong, Shanghai, 201321 China; 3 Department of Radiation Oncology, Shanghai Proton and Heavy Ion Center, Fudan University Shanghai Cancer Center, Shanghai, China

**Keywords:** Particle radiotherapy, Orbital malignancies, Eye-sparing surgery

## Abstract

**Background:**

To report the clinical experience of eye sparing surgery (ESS) and adjuvant carbon-ion or proton radiotherapy (CIRT or PRT) for orbital malignancies.

**Methods:**

An analysis of the retrospective data registry from the Shanghai Proton and Heavy Ion Center for patients with orbital tumors was conducted. The 2-year local progression-free, regional recurrence-free, distant metastasis-free, progression-free, and overall survival (LPFS, RRFS, DMFS, PFS, OS) rates as well as associated prognostic indicators were analyzed. Radiotherapy-induced acute and late toxicities were summarized.

**Results:**

Between 7/2014 to 5/2018, 22 patients with orbital malignancies of various pathologies received ESS followed by CIRT (18), PRT (1), or PRT + CIRT boost (3). With a median follow-up of 20.25 (range 3.8–38.8) months, the 2-year OS, PFS, LPFS, RRFS, and DMFS rates were 100, 57.9, 92.9, 93.3, and 72.8%, respectively. No acute severe (i.e., ≥grade 3) toxicity was observed. Two patients experienced severe visual impairment as late toxicities.

**Conclusion:**

With few observed acute and late toxicities, particle radiotherapy following ESS provided effective local control with infrequent severe toxicities for patients with orbital malignancies.

## Background

Orbital tumors are relatively rare with an incidence of 3.4/10^6^ person-years [1]; however, its management poses a major challenge to oncologists due to the complexities in the pathologies of the tumors and their proximity to the critical organs at risk (OARs).

Orbital malignancies can arise from any of the orbital structures such as extra-ocular muscles, fat, glands, vessels, nerves, and ocular adnexa. Extensive resection inevitably causes vision damage and disfigurement. Eye-sparing surgery (ESS) is the current preferred primary treatment for nearly all types of neoplasm of epithelial or mesenchymal origin [2]; nevertheless, sufficient margins are difficult to achieve especially for locally advanced diseases. Limited resection poses a high risk of local recurrence.

Multidisciplinary approach including surgery followed by adjuvant radiotherapy or chemoradiation is usually needed for orbital malignancies. Intensity-modulated radiotherapy (IMRT) has been used adjuvantly after surgery or in definitive settings for unresectable cases; however, radiation-induced toxicity limits the doses of IMRT to tumor targets due to excessive entrance and exit doses in the beam paths [3]. Lower doses are usually insufficient for controlling the more commonly diagnosed orbital malignancies including squamous cell carcinoma, adenoid cystic carcinoma (ACC) and soft-tissue sarcoma (STS) [1, 2, 4–6].

There is an increasing interest in the use of particle radiotherapy such as carbon-ion or proton radiotherapy (CIRT or PRT) in the management of head and neck malignancies, particularly for those occurred close to critical OARs, such as orbital tumors [7]. Due to its unique physical characteristic of Bragg Peak, particle radiotherapy allows for providing a high-dose coverage to the tumor with relatively low entrance and minimal exit doses [8, 9]. The use of intensity-modulated particle therapy (IMPT) technology may further improve dose distribution and reduce adverse-effects without compromising efficacy in the treatment of cancers within complex anatomical scenario thereby improves the therapeutic ratio [10, 11].

Carbon-ion beam has higher linear energy transfer (LET) and relative biological effectiveness (RBE) as compared to those of photon or proton [12–15]. The advantages in both physical and biological characteristics of carbon ion, in theory, make it more suitable in the management of conditions with both anatomic limitations and the radio-resistance such as ACC, melanoma, and sarcoma of the orbit. However, data describing clinical outcomes after particle radiotherapy especially CIRT for tumors of the orbit or ocular adnexa is lacking.

The Shanghai Proton and Heavy Ion Center (SPHIC) started to provide IMPT using pencil beam scanning (PBS) technology in 5/2015 [16]. In this article, we report the outcomes in terms of efficacy and safety of a group of patients with orbital tumors treated with adjuvant particle radiotherapy after ESS.

## Methods

### Pretreatment evaluation

Pretreatment evaluations included a complete history and physical examination (H&P), complete blood count (CBC), serum electrolytes, and MRI or CT (if MRI was contraindicated) of the head and neck region. PET-CT was performed if clinically indicated.

All patients were staged with the AJCC staging system (7th or 8th edition depend on the date of diagnosis). All protocols were registered to the institutional review board (IRB) of the SPHIC. All cases were discussed in the multidisciplinary tumor clinic of SPHIC to confirm the indication of adjuvant particle radiotherapy before inclusion into the institutional cancer registry and planning.

### IMPT and chemotherapy

All patients were immobilized with AlphaCradle® and thermoplastic masks in supine position. Plain CT for simulation from the vertex to the inferior margin of clavicular heads were performed at 1.5-mm slice thickness. MRI-CT fusion was performed for all patients prior to target delineation. The gross tumor volume (GTV) was defined as the tumor discovered on clinical examination or imaging studies for patients with incomplete surgical resection. We define clinical target volume (CTV) covering post-surgical GTV (CTV-G) after R2 resection/biopsy to deliver prescribed doses as GTV plus 1-3 mm margin (depend on the proximity to OARs). CTV for patients with R1 resection or achieved complete response (CR) after chemotherapy included pretreatment tumor bed plus high-risk areas for tumor extension. An additional 3–6 mm margin was added to the CTVs to create the planning target volume (PTV) for uncertainty with regard to dose distribution and potential setup errors.

Doses of particle radiotherapy were measured by Gy-equivalents (GyE) to account for the RBE differences compared to photon. Dose constraints of critical OARs are based on TD5/5 described by Emami et al. [17] except for optic nerve (D20 < 30GyE) and temporal lobes (V40 < 7.66 cc; V50 < 4.66 cc) set forth by the National Institute or Radiation Science of Japan [18]. For patients who had previous photon-based radiation, old treatment plans were obtained. Recovery from previous radiotherapy doses was set at 70% [19]. Planning for particle radiotherapy were performed using the Siemens Syngo® treatment planning system.

CIRT and PRT were delivered with PBS technology. Two-3 beams were typically delivered from the horizontal or 45^o^ directions. Setup accuracy was confirmed using bony landmarks on orthogonal X-ray on daily basis. Weekly CT were required to verify tumor regression/progression and anatomic changes. Chemotherapy was used at the discretion of the attending oncologists.

### Follow-up

All patients were admitted and examined daily during particle radiotherapy. After the discharge, all patients were encouraged to be followed-up using the standardized institutional follow-up protocol. The first follow-up was provided within 4–6 weeks after the completion of treatment. Patients were then followed-up every 3 months in the first 2 years, every 6 months in the following 3 years, and annually thereafter. A complete H&P with a focus to the eyes, orbits, head/neck region, as well as MRI of the head area are required at each follow-up. Other studies are ordered if clinically indicated.

### Data analysis

The duration of survival was calculated from the diagnosis of the disease until death or the last follow-up. The time to locoregional or distant failure was measured from the initiation of any treatment until disease progression or recurrence. Freedom from failure and OS rates were calculated using the Kaplan-Meier method. Cox regression model as was used for both uni- and multi-variate analyses to compare the difference of the survival probabilities and to define significant prognostic factors. All analyses were performed using the SPSS statistics package (Version 22.0).

Adverse events were scored by the attending radiation oncologist(s) according to the CTCAE (version 4.03). Acute toxicities included the adverse events occurred during or within 3 months after the initiation of particle radiotherapy. Late toxicity was defined as those occurred after 3 months from or persisted for > 3 months after the initiation of particle radiotherapy.

## Results

### Characteristics of patients and surgery

Between 11/2015 and 6/2018, 23 consecutive patients with orbital tumor were treated at SPHIC. All patients had ESS before particle radiotherapy. One patient was excluded from this analysis due to a change of diagnosis from pathology confirmation in the mid of CIRT which substantially changed treatment. The median follow-up of the remaining 22 patients was 20.25 (range 3.8–38.8) months.

Most patients (81.8%) presented with malignancies of the lacrimal gland or lacrimal sac, and 77.2% had malignancies of epithelial origin. One patient presented with locally recurrent lacrimal gland ACC had ESS twice. She also received photon-based radiotherapy (60Gy/30Fx) after the first surgery. The characteristics of the patients, their diseases, and treatment were detailed in Table 1.

**Table 1:** Characteristics of patients, their disease, and treatment

Characteristic	No. of patients (%)
Median age (range)	46.5 (14–74)
Sex	
Male	14 (63.6)
Female	8 (36.4)
Tumor site	
Lacrimal gland	13 (59.1)
Lacrimal sac	5 (22.7)
Orbital bone	1 (4.5)
Other	3 (13.6)
Tumor histology	
Adenoid cystic carcinoma	11 (50.0)
Adenocarcinoma	5 (22.7)
Squamous cells carcinoma	1 (4.5)
Melanoma	1 (4.5)
rhabdomyosarcoma	1 (4.5)
desmoplastic small round cell tumor	1 (4.5)
alveolar soft part sarcoma	1 (4.5)
chondrosarcoma	1 (4.5)
T category	
T1	2 (9.1)
T2	7 (31.8)
T3	4 (18.2)
T4	9 (40.9)
Tumor status	
Primary	21 (95.5)
Recurrence	1 (4.5)
Surgical margin	
R0	3 (13.6)
R1	6 (27.3)
R2 or biopsy	13 (59.1)
Interval from surgery to radiotherapy, mo	
Median (range)	2.2 (1.2–6.13)
Radiotherapy technique	
PRT	1 (4.5)
CIRT	18 (81.8)
PRT + CIRT	3 (13.6)
Radiotherapy dose (Gy BED)	
Median (range)	85.05 (67.2–94.5)
GTV (ml)	
Median (range)	16.0 (1.9–67.6)
CTV (ml)	
Median (range)	43.4 (18.8–209.9)
Concurrent chemotherapy or immunotherapy	
Cisplatin	2 (9.1)
Interferon α-2b	1 (4.5)
No	19 (86.4)

### Particle radiotherapy

PRT, CIRT, or their combination were used in 1, 18, and 3 patients with curative intention, respectively. The median time between surgery and particle therapy was 2.2 months (range 1.2–6.13).

**Fig. 1:**
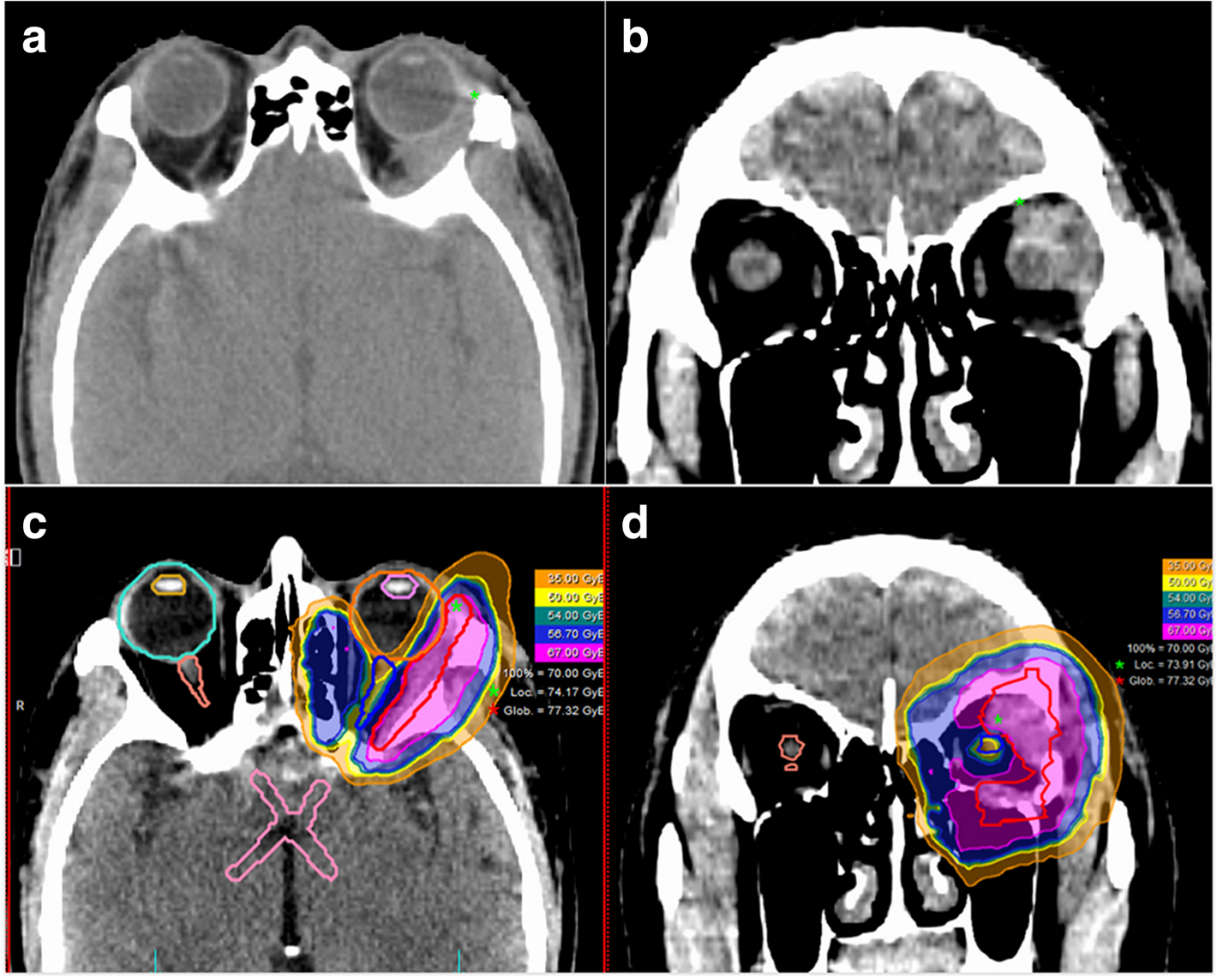
Axial (**a**) and coronal (**b**) views of a post eye sparing surgery CT scan of a patient with left lacrimal gland ACC. Axial (**c**) and coronal (**d**) views of a typical intensity-modulated carbon ion radiotherapy treatment plan

Three patients who achieved R0 resection received PRT (56 GyE/28 fractions, 1 case) or CIRT (60 GyE/20 fractions, 2 cases), respectively. For the remaining 19 patients, 3 received PRT (56 GyE/28 fractions) followed by CIRT boost (15 GyE/3 fractions), and 16 received CIRT (60–70 GyE for primary/residual tumor and 54-62GyE for CTVs in 18–23 fractions using simultaneous integrated boost technique). Elective nodal irradiation was not performed for any patients. All 22 patients completed particle radiotherapy without break. A typical treatment plan is illustrated in Fig. 1.

### Survival outcomes

**Fig. 2:**
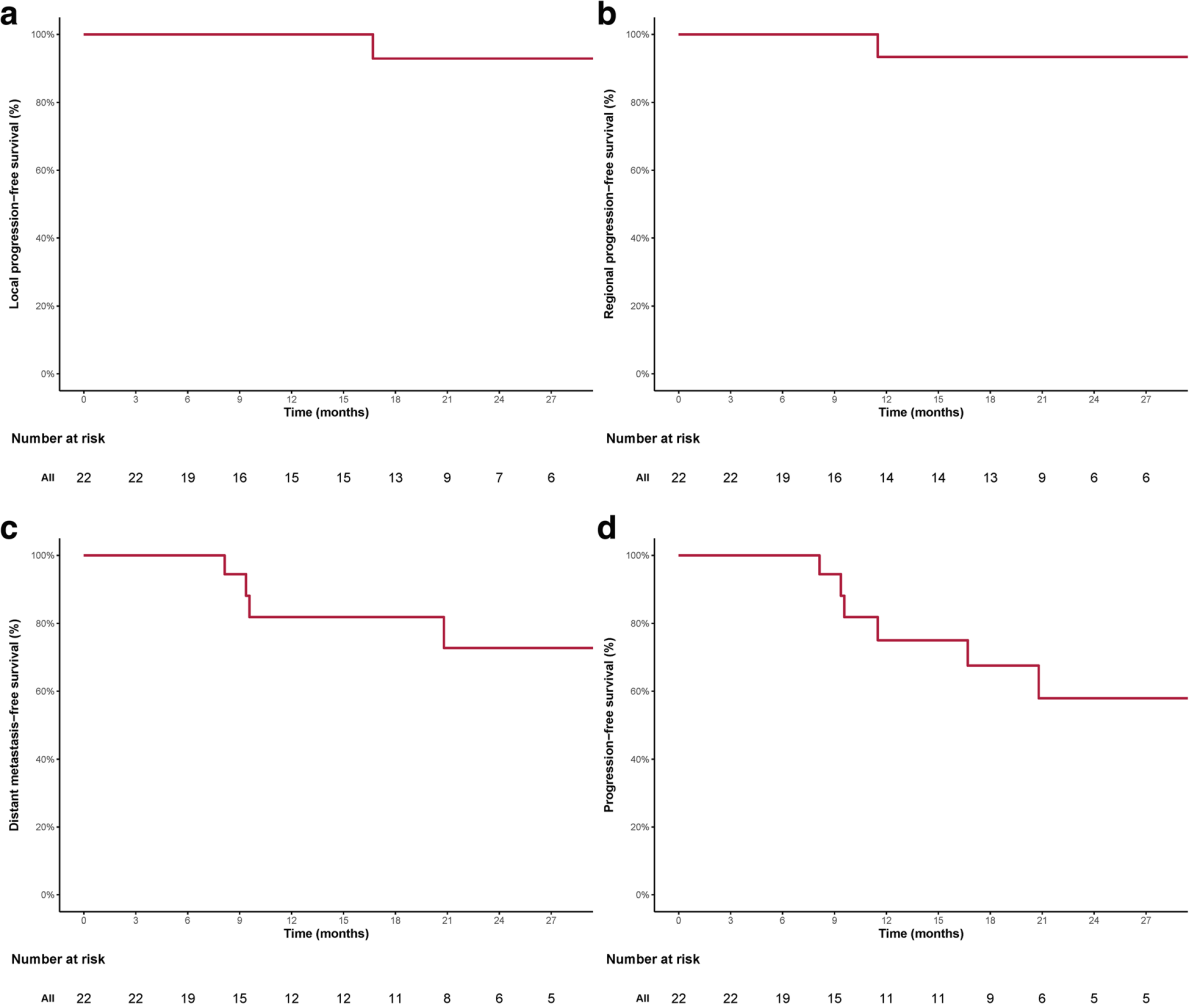
Local progression-free survival (LPFS) (**a**), regional recurrence-free survival (**b**), distant metastasis free-survival (DMFS) (**c**), and progression-free survival (PFS) (**d**) rate curves of the entire cohort

With a median follow-up of 20.25 (range 3.8–38.8) months, all 22 patients were alive. One patient who had R2 resection followed-by cisplatin chemotherapy for T3N0M0 rhabdomyosarcoma of the lacrimal sac developed local recurrence at 17.8 months after CIRT. Another with R2 resected T4N0M0 ACC of the lacrimal gland developed regional recurrence after PRT + CIRT boost at 11.5 months. The 2-year local-progression-free and regional-recurrence-free survival (LPFS and RRFS) rates were 92.9 and 93.3%, respectively (Fig. 2a & b). Four patients with lacrimal gland malignancies (2 with ACC and 2 with adenocarcinoma, 2 with T2 and 2 with T4 disease) developed distant metastasis (DM) at a median time of 9.47 months (range 8.13–20.8). The 2-year DM-free survival (DMFS) rate was 72.8%, and the 2-year progression free survival (PFS) rate was 57.9% (Fig. 2c & d) for the entire cohort. None of the 13 patients with lacrimal gland malignancy developed local failure or progression (i.e., LPFS = 100%). The 2-year RRFS, DMFS and PFS were 88.9 and 53.0% and 39.3% for patients with lacrimal gland malignancies, respectively.

### Acute and chronic toxicities

The characteristics of acute and late toxicities are summarized in Table 2. Nine patients (40.9%) experienced grade 1 or 2 acute toxicities induced by particle radiotherapy. No acute toxicity of grade 3 or above was observed. Seven patients (31.8%) experienced late toxicities of various grades including 3 with grade 1 dry eyes, 1 with grade 1 brain injury, 1 with grade 2 retinopathy, 1 with grade 3 visual impairment and 1 with blindness in the affected eye (grade 4). During the follow-up period, no vision impairment was observed, except for 2 patients developed grade 3 and grade 4 visual acuity reduction after CIRT. One of them experienced ipsilateral vision acuity reduction from normal to 20/200–40/200 at 6 months accompanied by optic atrophy diagnosed by MRI; the other patient developed blindness at 3 months without changes on MR scan. One patient who has affected eyeball fixation due to twice eye-sparing surgery and photon-based radiotherapy prior to re-irradiation by CIRT, and no ocular movement disorder was observed in remaining patients who received particle radiotherapy. In addition, there was no eye injury in contralateral side in all patients at present.

**Table 2:**
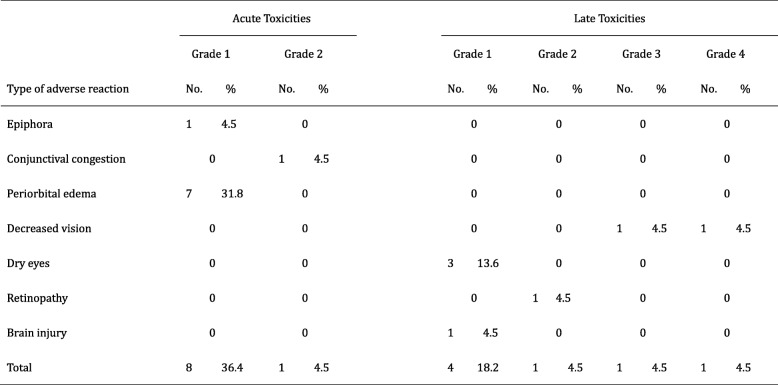
Characteristics of toxicities

### Prognostic factors

**Fig. 3:**
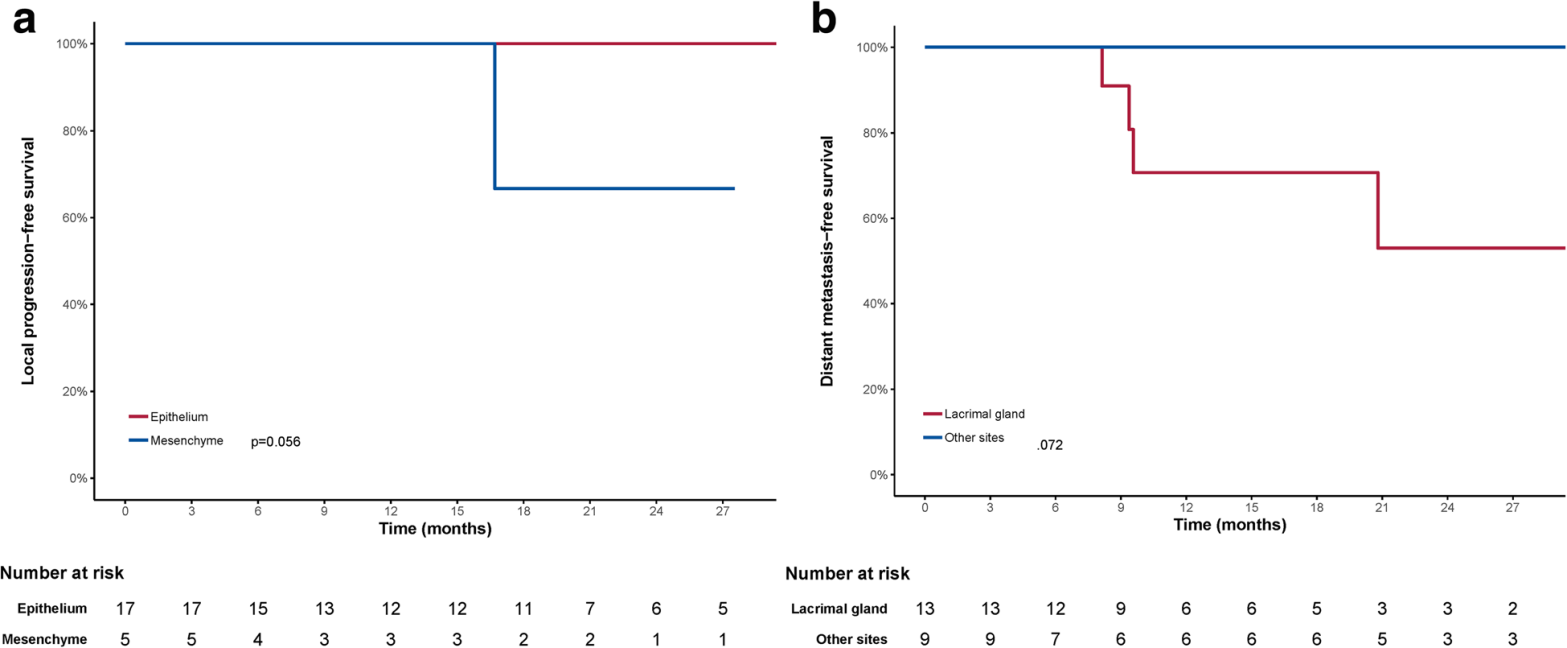
Local progression-free survival (**a**), Distant metastasis-free survival (**b**) curves showing malignancies of mesenchymal origin had a trend toward a worse LPFS and a trend that lacrimal gland tumor had worse DMFS

Univariate analyses using log-rank test indicated tumors with mesenchymal origins had a trend toward a worse LPFS (*p* = 0.056) (Table 3, Fig. 3a). In addition, tumors of lacrimal gland had a trend with worse DMFS (*p* = 0.072) (Table 3, Fig. 3b). When BED was taken as a continuous variable using Cox regression analysis, higher BED had a trend to associate with improved DMFS (hazard ratio, 0.884; 95% CI, 0.776–1.007 [*P* = 0.064]) (Table 4). However, margin status, T-classification, volume of GTV or CTV did not associate with PFS, LPFS, RRFS or DMFS in both log-rank test and Cox regression analysis (Tables 3 & 4).

**Table 3:** Univariate analysis by the log-rank test

Characteristics	PFS	LPFS	RRFS	DMFS
Gender	0.910	0.527	0.480	0.362
Age (<46.5 y vs. >46.5 y)	0.442	0.527	0.480	0.847
Tumor site (other vs. lacrimal gland)	0.118	0.248	0.414	0.072
Origin (mesenchymal vs. epithelial histology)	0.653	0.056	0.617	0.254
T classification (T1/2 vs. T3/4)	0.524	0.386	0.414	0.784
Surgical margin (R0 + R1 vs. R2)	0.887	0.317	0.350	0.221
BED (<85.05 GyE vs. ≥85.05 GyE)	0.813	0.602	0.617	0.806
GTV (< 16 ml vs.>16 ml)	0.928	0.527	0.157	0.666
CTV (< 43.4 ml vs.>43.4 ml)	0.431	0.317	0.285	0.327

**Table 4:** Univariate analysis of DMFS and PFS by Cox regression

	DMFS		PFS	
Variables	P	HR (95% CI)	P	HR (95% CI)
Gender				
Male	Ref	Ref	Ref	Ref
Female	0.378	2.431 (0.338–17.48)	0.910	1.103 (0.202–6.036)
Age, y	0.186	1.046 (0.979–1.118)	0.752	1.001 (0.958–1.061)
Histology				
Epithelium	Ref	Ref	Ref	Ref
Mesenchyme	0.488	0.031 (0.001–560.47)	0.656	0.613 (0.071–5.280)
T classification				
T1 + T2	Ref	Ref	Ref	Ref
T3 + T4	0.784	0.760 (0.107–5.415)	0.529	1.731 (0.313–9.564)
Surgical margin				
R0 + R1	Ref	Ref	Ref	Ref
R2	0.254	0.268 (0.028–2.579)	0.887	0.890 (0.178–4.450)
BED	0.064	0.884 (0.776–1.007)	0.100	0.898 (0.791–1.021)
GTV, ml	0.948	1.002 (0.948–1.059)	0.928	0.998 (0.953–1.045)
CTV, ml	0.978	1.000 (0.980–1.021)	0.797	0.998 (0.980–1.016)

Multivariate analysis using Cox regression using tumor correlation factors such as histological type, BED (continuous variable), T-classification, volume of GTV and CTV suggested a significant relationship for higher BED with improved PFS (hazard ratio, 0.732; 95% CI, 0.557–0.960 [*P* = 0.024]) and a trend with improved DMFS (hazard ratio, 0.717; 95% CI, 0.512–1.004 [*P* = 0.053]). Furthermore, larger CTV field may improve PFS (hazard ratio, 0.952; 95% CI, 0.906–1.001 [*P* = 0.054]) (Table 5).

**Table 5:** Multivariate analysis of DMFS and PFS by Cox regression

	DMFS		PFS	
Variables	P	HR (95% CI)	P	HR (95% CI)
T classification				
T1 + T2	Ref	Ref	Ref	Ref
T3 + T4	0.368	6.673 (0.107–415.609)	0.189	6.28 (0.406–97.134)
Surgical margin				
R0 + R1	Ref	Ref	Ref	Ref
R2	0.233	0.042 (0–7.646)	0.969	1.059 (0.059–18.20)
Histology				
Epithelium	Ref	Ref	Ref	Ref
Mesenchyme	0.976	0	0.384	0.255 (0.012–5.548)
BED	0.053	0.717 (0.512–1.004)	0.023	0.726 (0.551–0.957)
GTV, ml	0.079	1.199 (0.979–1.469)	0.104	1.117 (0.977–1.001)
CTV, ml	0.095	0.946 (0.887–1.010)	0.053	0.953 (0.907–1.278)

## Discussion

We analyzed 22 patients with orbital tumor after ESS followed by PRT and/or CIRT. With a median follow-up of 20.3 months, the 2-year OS, PFS, LPFS, RRFS, and DMFS rates were 100, 57.9, 92.9, 93.3, and 72.8%, respectively. No acute severe (i.e., ≥grade 3) toxicity was observed. The occurrences of severe late toxicities were also infrequent. These findings suggest that particle radiotherapy after ESS could provide satisfactory local control with acceptable toxicities at 2 years for patients with orbit tumors. However, DM remains a challenge for overall disease control.

Due to the complexity of the anatomy, ocular exenteration was advocated histologically; however, disease control remained suboptimal. In a report of 39 patients with orbital malignancies received exenteration with (10 patients) or without (29 patients) adjuvant radiation, ~ 20% experienced local recurrence after a median follow-up of 34.7 weeks. The 3-year OS and recurrence/death-free survival rates were 50.5 and 47.5%, respectively [20]. Results from multiple retrospective studies revealed that 5-year LPFS ranged at 20–22% after surgery (exenteration or eye spearing) without adjuvant radiation [21, 22]. Adjuvant radiotherapy following exenteration produced substantially improved local control as compared to surgery alone. Three- or 5-year LPFS rates of 60~65% [21, 23], with similar OS rates of 60% have been reported. In a more recently published series, adjuvant IMRT after exenteration produced a 3-year LPFS and OS rates of 91 and 70%, respectively [24].

ESS provides an important opportunity for function preservation for patients with orbital tumors. Disease control and survival rates after less aggressive (i.e., eye-sparing) surgery assimilates those from exenteration when adjuvant radiotherapy was added. In 11 lacrimal gland tumor patients treated with ESS, only 1 patient declined adjuvant radiotherapy and developed local recurrence [25]. A more recently published series of 37 patients with lacrimal gland carcinoma (> 80% with T1 or T2 diseases) reported a 2-year recurrence free survival of approximately 95%. Of the 31 patients received adjuvant radiotherapy, 12 had PRT [22]. Although the composition of patients and pathologies in our series differ substantially from the above-mentioned papers, the outcomes remain encouraging. All 22 patients were alive at the time of analysis with a 2-year LPFS rate of 92.9%, although ~ 60% of patients in our series presented with T3/T4 diseases. Furthermore, patients with lacrimal gland tumors achieved 2-year LPFS and RRFS rates of 100 and 88.9%, respectively, although ~ 40% had T4 disease. Our results mimicked the most favorable 2-year outcome in terms of survival and local control despite of a less favorable clinical presentation [21, 24, 26, 27].

With effective locoregional control, DM became the most common mode of failure in patients with orbital malignancies. DM rate of 27.5% and 3-year DMFS of 70% were reported for orbital carcinomas [23]. However, DM is more challenging for lacrimal glade cancer especially adenoid cystic carcinoma [21, 23]. Skinner et al. [21] reported a 5-year DMFS rate of 65% for lacrimal gland carcinoma patients, and found that DM was not correlated with histopathology, surgical margin or type of surgery (eye-spearing vs. exenteration). These results were in line with our finds, of which DM was seen in 30.8% of lacrimal gland patients. Furthermore, the 2-year DMFS of our entire cohort and those with lacrimal gland malignancies were 71.6 and 53%, respectively. Our univariate analysis indicated a trend for DM in patients with lacrimal gland malignancies (*P* = 0.072). In both the univariate and multivariate analysis, factors such as histopathology, surgical margin status, or T-classification were not associated with DMFS. However, BED may have significant impact on DMFS: The higher the BED, the lower the risk of DM. Moreover, the overall PFS, largely related to DMFS, is not only significantly associated with BED but also may correlated with CTV volume (*P* = 0.053). These findings suggested that particle therapy may have a significant impact on disease control due to its improved conformality secondary to its physical characteristics. Well localized and more precise dose distribution enables higher dose as well as bigger CTV with OAR sparing, a feature that is important in particle radiotherapy for most head and neck cancers [10, 11]. Furthermore, CIRT not only provide advantages in dose distribution, but also biologically due to its higher linear energy transfer (LET). The value of the RBE of carbon-ion is 2–5:1 as compared with photons and protons, which is highly relevant for radio-resistant tumors such as STS [14, 15]. Our data suggested mesenchymal malignancies of the orbit may pose a higher risk of local recurrence (*p* = 0.056). And our previous experience with CIRT for head and neck sarcomas revealed favorable disease control with acceptable toxicity profile [28].

In spite of the improved function preservation and locoregional disease control with ESS and adjuvant IMRT, radiation-induced toxicities remained a challenge in the management of orbital tumors due to its anatomical complexity [29, 30]. Published data indicated that with doses exceeding 50Gy, conjunctival keratinization, lacrimal gland atrophy and fibrosis, corneal decompensation would occur. When doses exceeded 60Gy, symblepharon, keratoconjunctivitis, permanent dry eyes became a concern. Moreover, the probability of radiation-induced optic neuropathy may occur in 7–20% of patients [6, 17, 31, 32]. Particle radiotherapy provides distinctive advantages for tumors close to critical OARs such as orbital malignancies due to its distinctive physical characteristics. Patterns of treatment-induced adverse effects were studied extensively in a series of 20 orbital tumor patients treated with ESS followed by PRT at M.D. Anderson Cancer Center [7]. Although disease control was not the focus of the study, the authors reported one patient with local and another with regional recurrence. In addition to the 35% patients who experienced grade 3 acute dermatitis, 30% experienced grade 3 chronic toxicities of epiphora and eyelid function disorder. In addition, grade 2, 3, and 4 visual decrease were observed in 2, 2, and 1 patient, respectively. The risk of severe chronic toxicity was higher when the maximum corneal dose exceeded 36Gy (BED) [7]. Higher BED was found to associate with improved outcome in our series, indicating the advantage of particle radiotherapy for this condition which usually occur close to dose limiting OARs. CIRT with less penumbra as compared to proton may provide additional physical advantage. In addition, favorable outcomes in terms of radiation-induced toxicities were observed in our series: Only grade 1/2 acute adverse-effects were observed in 9 patients (40.9%). Approximately 22.7% of patients developed grade 1 or 2 late effects. Nevertheless, 2 patients experienced severe decrease of vision at 3 and 6 months after CIRT. In both cases the tumors were attached or close to the optic nerve or eye.

Several pitfalls of our study need to be discussed. First, because of variations in certain institutional clinical trial regimens, few patients were treated with PRT (1 cases) or PRT + CIRT boost (3 cases) although most patients received CIRT alone. Our analysis largely reflected the results after CIRT for orbital malignancies. Second, orbital tumor includes a group of heterogenous conditions from various origins with substantial different biological behaviors. Combining different pathologies would inevitably affect the uniformity of the results. Third, owing to the limited follow up time, these clinical results must be considered with caution, we will continue to follow up these patients and report clinical outcomes with longer period. Fourth, our study suffered from the nature of retrospective studies with a relatively small sample size; nevertheless, we provided the outcomes of the largest series of orbital tumors treated with particle radiotherapy in terms of disease, control, survival, and safety. Considering the rarity of the condition, nearly all published literatures were retrospective in nature from single institutions. Clearly, prospective investigations to compare efficacies from different treatment modalities or technologies are difficult to initiate without international collaboration among specialized academic centers.

## Conclusion

Adjuvant particle radiotherapy following ESS provided a satisfactory OS and locoregional control at 2 years. DM remained a major form of treatment failure. No severe acute treatment-induced toxicity was observed, and severe late toxicities was observed in < 10% of cases. Long-term follow-up is needed to confirm the efficacy and safety of adjuvant particle radiotherapy, in particular CIRT, for orbital tumor after ESS.

## Data Availability

The datasets used and/or analyzed during the current study are available from the corresponding author on reasonable request.

## Abbreviations

ACC: Adenoid cystic carcinoma
BED: Biological equivalent dose
CBC: Complete blood count
CIRT: Carbon-ion radiotherapy
CR: Complete response
CT: Computed tomography
CTCAE: Common terminology criteria for adverse events
CTV: Clinical target volume
CTV-G: Post-surgical GTV
DMFS: Distant metastasis free survival
ESS: Eye sparing surgery
GTV: Gross tumor volume
GyE: Gy-equivalents
H&P: History and physical examination
IMPT: Intensity-modulated particle therapy
IMRT: Intensity-modulated radiotherapy
IRB: Institutional review board
LET: Linear energy transfer
LPFS: Local progression-free survival
MRI: Magnetic resonance imaging
OARs: Organs at risk
OS: Overall survival
PBS: Pencil beam scanning
PET-CT: Positron emission tomography/computed tomography
PFS: Progression free survival
PRT: Proton radiotherapy
PTV: Planning target volume
RBE: Relative biological effectiveness
RRFS: Regional recurrence-free survival
SPHIC: Shanghai Proton and Heavy Ion Center
STS: Soft-tissue sarcoma
